# A Perspective on Inhabited Urban Space: Land Use and Occupation, Heat Islands, and Precarious Urbanization as Determinants of Territorial Receptivity to Dengue in the City of Rio De Janeiro

**DOI:** 10.3390/ijerph17186537

**Published:** 2020-09-08

**Authors:** Jefferson Pereira Caldas Santos, Nildimar Alves Honório, Christovam Barcellos, Aline Araújo Nobre

**Affiliations:** 1 Centro de Inovação em Biodiversidade e Saúde, Instituto de Tecnologia em Fármacos, Fundação Oswaldo Cruz, Rio de Janeiro 22775-903, Brazil; 2 Laboratório de Mosquitos Transmissores de Hematozoários, Instituto Oswaldo Cruz, Fundaҫão Oswaldo Cruz, Rio de Janeiro 21040-900, Brazil; nildimar.honorio@ioc.fiocruz.br; 3 Núcleo Operacional Sentinela de Mosquitos Vetores-Nosmove/Fiocruz, Fundaҫão Oswaldo Cruz, Rio de Janeiro 21040-900, Brazil; 4 Instituto de Comunicação e Informação Científica e Tecnológica em Saúde, Fundação Oswaldo Cruz, Rio de Janeiro 21040-900, Brazil; xris@icict.fiocruz.br; 5 Programa de Computação Científica, Fundação Oswaldo Cruz, Rio de Janeiro 21040-900, Brazil; aline.nobre@fiocruz.br

**Keywords:** arbovirus, dengue, receptivity, territory, Rio de Janeiro

## Abstract

Introduction: Rio de Janeiro is the second-largest city in Brazil, with strong socio-spatial segregation, and diverse and heterogeneous land use, occupation, and landscapes. The complexity of dengue requires the construction of surveillance and control tools that take into account the historical, social, economic, and environmental processes mediated in the territory as a central axis of public policy. In this context, this study aimed to stratify the city into areas of receptivity to dengue, using innovative “territorial indicators” because they are built based on the actual occupation of the territory. Methods: We designed and constructed 17 indicators that sought to characterize the transformed and inhabited space according to receptivity to dengue. We used data on land use and occupation, connectivity, climate, and landscape. We developed the dengue receptivity through principal component analysis (PCA), using multiple criteria analysis and map algebra integrated in a GIS platform. Results: The most receptive areas were concentrated in the transition between the north and west zones of the city, a region of unconsolidated urban sprawl. The areas of greatest receptivity had the highest incidence and density of *Aedes* eggs during the study period. The correlation between receptivity index and incidence rate was positive in the epidemic years. Conclusion: The proposed set of indicators was able to identify areas of greater receptivity, such as regions of disorderly urban sprawl, with a concentration of social and environmental processes that are related to the occurrence of dengue outbreaks and high vector density. On the other hand, population immunity plays an important role in the spatial distribution of dengue during non-epidemic years.

## 1. Introduction

Arboviruses are severe global public health problems because of their high rates of morbidity and mortality, and their encroachment into new geographical areas [1,2,3,4]. Environmental changes through anthropogenic action, disorderly urban growth, globalization, and climate variability are space-shaping vectors that create favorable ecological conditions for the emergence and spread of vector-borne human infectious diseases, such as dengue, chikungunya, Zika and yellow fever [2,3,5,6].

Dengue is the most prevalent arbovirus in the world, with an estimated 390 million annual cases. The incidence rates of this arbovirus have been increasing around the world, following the territorial expansion of transmission areas [7]. After the introduction of the virus, the establishment and spread of dengue epidemics in cities depend on the conformation and occupation of the territory, and human mobility and population immunity [8]. Therefore, the recognition, description, and detailed representation of the territory is an essential step in epidemiological studies on the dynamics of disease transmission. However, the complexity of the territory is often reduced to a set of poverty and social inequality indicators, wiping the combined effect of place and population.

The city is configured as a heterogeneous, fragmented, and dispersed space, with different uses and social contents. The city is connected through economic networks, linking the upper circuit of the urban economy, based on high technology and information flow, with the lower circuit based on low technology and intensive, often informal labor [9].

Currently, cities are vast social and environmental mosaics of interconnected places, with different local realities expressed in space, promoting particular conditions for the production and reproduction of different diseases. In population health this also occurs in different ways in space and time, involving a complex chain of situations and events [10,11]. According to Barcellos et al. (2002) [12], disease can be considered an individual manifestation; however, the health situation is a manifestation of the lived and inhabited space.

In epidemiological studies, the concepts of vulnerability and receptivity have been used to express and characterize an area or population group in terms of the risk of epidemics and outbreaks of a disease [13]. These two concepts are widely used in malaria studies, in which vulnerability refers to the likelihood of an infected individual or vector originating from endemic areas introducing the parasite into other receptive areas. On the other hand, receptivity refers to the presence of ideal conditions for disease transmission, namely, the existence of a minimum density of competent vectors and the existence of ecological and climatic factors that favor transmission [13,14].

Site, situation, and function are concepts from urban geography associated with the occurrence of urban epidemics. This is because both natural and society-constructed spaces are shaped by a complex and dynamic network of fixed objects and flows within a system in which space and social process are in continuous interaction [15]. This process results in different realities of receptivity and vulnerability to arboviruses due to the conformation of geographic network of causalities required for the establishment of a particular disease in the territory.

Vulnerability depends on the function and situation resulting mainly from the flows affecting this area. It therefore responds more to situational and dynamic processes operating in the territory. On the other hand, the concept of receptivity is understood in this work as a result of the association between the concepts of site and situation and the concept of fixed objects, thereby representing the capacity of a given site to receive and maintain the situation of arbovirus transmission based on the structure that anthropic natural fixes confer to the territory in question [16,17]. 

Rio de Janeiro is the second-largest city in Brazil, marked by strong social and spatial segregation and diversity of land use and occupation, and of landscapes. Its site is characterized by the presence of Atlantic forest remnants, the influence of the Atlantic coast, and its rugged relief. Within the city, these conditions can produce places with greater or lesser receptivity to the introduction and maintenance of dengue in the urban space. Historically, the city of Rio de Janeiro has experienced successive dengue epidemics, playing an essential role in most major dengue epidemics in the country and in the introduction and dispersion of new serotypes [18,19,20].

From the 1980s onwards, the concept of territory was considered a central category in the operationalization of health surveillance actions aimed at observing and contextualizing the social and environmental determinants of health [21]. However, the concept is most often underutilized due to its exclusive use as an administrative-political division to represent space through indicators from the Demographic Census [22]. In general, no refinement is sought in the production of data that expresses the actual pattern of use, occupation, and complexity that shapes the territory. With the advances in geoprocessing and remote sensing techniques, it is possible to delimit and represent other processes present in the territory, according to socio-environmental determinants of the issue to be studied, such as the presence of heat islands, vegetation cover, and spatial mobility, among other factors, generally disregarded in spatial analyses of dengue.

The areas with the highest rates for arboviruses in Brazil and Rio de Janeiro are generally concentrated in the same territories where dengue has been present for many years. This highlights the possible failure of current strategies for addressing arboviruses, which are focused on combating *Aedes aegypti*, not considering the social, environmental, and territorial determinants of health [23]. Therefore, it is essential to build strategies for addressing arboviruses that oppose this model, while understanding that the health-disease process results from historical, social, and environmental processes mediated in the territory. Health surveillance should have a territorial basis, incorporating social and environmental determination as a guiding axis.

In this context, this study aimed to stratify the city of Rio de Janeiro into areas of dengue receptivity through innovative “territorial indicators” whose construction takes into account the actual reality of the occupation of the territory. 

## 2. Methods

### 2.1. Study Area

**Figure 1:**
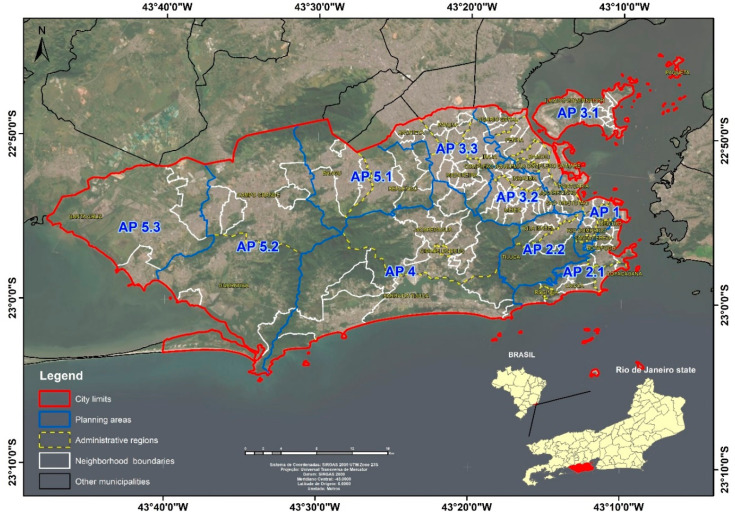
Location map of the city of Rio de Janeiro with its political and administrative divisions.

The city of Rio de Janeiro, capital of the state of Rio de Janeiro, is situated at 22°54′23″ latitude south and 43°10′21″ longitude west. Located in the southeastern region of the country, it has an area of approximately 1197 km^2^ and a population of 6,320,446 inhabitants in 2010 [24]. It has significant spatial heterogeneity generated by the process of urban land use and occupation, and its peculiar physiographic characteristics, making this city a mosaic of contrasting urban and environmental landscapes [25,26]. It is politically divided into 160 neighborhoods and 33 administrative regions (Figure 1).

### 2.2. Study Design

We carried out a territory-based ecological study taking the neighborhood as a spatial unit of analysis. This scale was chosen due to the ease in obtaining and aggregating socioeconomic and epidemiological data and because it is recognized by the population in its daily use.

### 2.3. Construction of Indicators

We designed and constructed 17 indicators based on the social and environmental determinants of dengue (Table 1). These indicators seek to characterize the inhabited space of the city and its receptivity to dengue. The indicators were aggregated by the 160 neighborhoods of the city of Rio de Janeiro, considering only their portions that are effectively occupied by a human population, with the objective of better portraying the territorial reality of the municipality.

The area of the city that is effectively occupied consists of areas with anthropic constructions. We initially used the methodology of “supervised classification” of a Landsat 8 (United States Geological Survey (USGS)) satellite image, later refined by visual interpretation, to map this area for each neighborhood. This interpretation consists of the manual vectorization of the classes of interest.

In the process of refining the classification, we delimited the residential area within the effectively-occupied area (excluding park areas, uninhabited mountains, and bodies of water), by excluding areas of public facilities such as schools, cemeteries, the airport, and the port, among others. These two mappings were used as a basis for building most of the indicators used in this study. 

**Figure 2:**
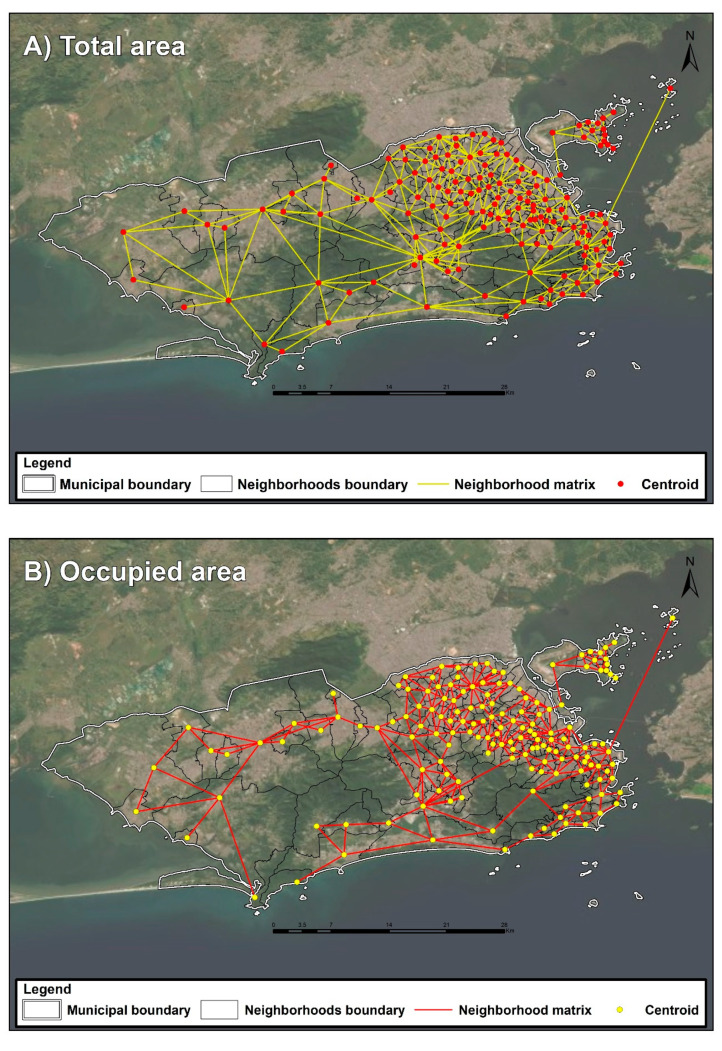
Matrices of neighborhood proximity, based on (**A**) total area and (**B**) occupied area of the city of Rio de Janeiro.

Two spatial connectivity matrices—also known as neighborhood matrices—were constructed, both based on contiguity. The first considered the cartographic grid of administrative and political neighborhoods, and the second took into account the cartographic grid of the neighborhoods’ occupied areas (Figure 2). The matrix constructed based on the occupied area was used to build two indicators that more adequately reflect the connections between the neighborhoods and the resulting mobility of population among neighborhoods.

Data regarding land use and occupation, neighborhood matrix, climate, and the city landscape were used for the construction of the indicators. In this way, we sought to consider the complexity of the dengue health-disease process in the city of Rio de Janeiro.

Based on the mapping of the effectively-occupied area, we constructed the indicator “percentage of the effectively-occupied area in relation to the total area of the political and administrative limits of each neighborhood” (Ind 01). Based on the mapping of the residential area, we constructed the indicator “percentage of the residential area in relation to the total area of each neighborhood” (Ind 02). We calculated the “net demographic density” (Ind 03) based on the ratio of the total population residing in each neighborhood to the residential area of each neighborhood. These indicators are associated with how the territory is occupied by human populations. Hypothesized that areas with higher percentage of human occupation and demographic density are areas with higher risk for the spread of dengue due to the greater proximity between hosts and greater number of breeding sites available for *Aedes* vectors [27].

We considered areas of irregular occupation, with a high density of makeshift residential buildings as subnormal agglomerates. In Rio de Janeiro, most of these areas are slums (*favelas*) [24]. We calculated the percentage of subnormal agglomerate area in relation to the residential area of each neighborhood (Ind 04). To calculate this indicator, we updated the IBGE and municipal government cartographic bases of subnormal agglomerates [28,29] by actively searching locations with characteristics of *favelas* based on the visual interpretation of satellite images. The hypothesis that supports this indicator is that the more “favelized” areas, the greater the propensity of dengue dissemination is due to a greater condition of *Aedes* vectors proliferation and the greater ease of dengue spread due to the urban architecture of the slums where houses with precarious housing and sanitation conditions and people are very close.

We delimited strategic points (hotspots of potential vector breeding) such as cemeteries, tire services, warehouses, and other establishments that represent potential macro-foci of *Ae. aegypti* through georeferencing of the address database of these establishments provided by the municipal government, and subsequent vectorization of the area around each point through visual interpretation of satellite images. With this cartographic grid, we calculated the percentage of the area of strategic points in relation to the occupied area of each neighborhood (Ind 05). It deals with the percentages of areas of so called “strategic points” (cemeteries, rubbers, scraps, recycling, etc.) that are considered possible macro breeding sites available for *Aedes* vectors. Thus, the hypothesis that supports this indicator is that the higher the percentage of the area classified as a strategic point, the greater the risk for dengue [30].

We built the mean verticalization indicator by calculating the mean altitudes of all buildings per neighborhood (Ind 06)—the average value of the height of the residences of each neighborhood. This indicator was used because areas with higher verticalization are generally areas with higher income. With this, the hypothesis that underpinned this indicator was that in places with higher verticalization where income is higher and the backyards lower, the risk for dengue is lower [31].

Data on vegetation areas were obtained by mapping land use through the visual interpretation method. For this, we used a satellite image with a spatial resolution of 0.5 m. We classified vegetation into different classes and later calculated the percentage of area covered by vegetation (Ind 07), excluding parks and squares in relation the total neighborhood area. The hypothesis for the construction of this indicator was that in areas with a higher proportion of vegetated areas the risk for dengue is lower. Since you are usually areas are periurban and with low population density [32].

We calculated the number of connections in each neighborhood (Ind 08) based on contiguity, considering the cartographic grid of the occupied area. For each neighborhood, we calculated the length of the border (Ind 09) with other neighborhoods based on the cartographic grid of the occupied area. These indicators quantify the urban connection of each neighborhood. The hypothesis that sustains this the choice and construction of them was that neighborhoods greater urban connection and population transit facilitate dengue spatial diffusion [33].

We obtained surface temperature data from Modis satellite imagery for the period 2008–2014 [34]. Daytime and nighttime values, obtained in raster format, with cells of 1000 m by 1000 m, were aggregated according to the limit of the occupied areas of the neighborhoods, and we calculated their mean values for the entire period (Ind 10 and Ind 11, respectively). Rainfall data were obtained from the 33 rainfall gauging stations installed in the city and were grouped by month [35]. We georeferenced the stations and interpolated rainfall data using the Inverse Distance Weighting (IDW) method. We calculated the rainfall means for each neighborhood (Ind 12) using map algebras, considering only the occupied area for the entire study period. Indicators 10, 11 and 12 refer to the climatic conditions in specific temperature and precipitation averages for each neighborhood. The hypothesis that supported the choice of these indicators is that high temperatures and rainfall favor the development of *Aedes* vectors and consequently the risk for dengue [32,36].

We built indicators that characterize the landscape where households are located based on information about the environment available in the 2010 Census (Ind 13, Ind 14, Ind 15, Ind 16, and Ind 17). The rate for each neighborhood-level indicator was calculated by aggregating information according to census tract. All calculations used the number of households referring to each indicator as the numerator, and the total number of households as the denominator. They were constructed seeking to portray the conditions of urban infrastructure surrounding the households. Additionally, the hypothesis that supports these indicators is that in areas with less urban infrastructure the risk for dengue is higher [37,38,39].

In this study, we considered two outcomes to assess the relationship, through spatial overlap, with the receptive areas: incidence of dengue cases and *Aedes* egg density index (EDI).

We calculated the annual dengue incidence rate for each neighborhood through the ratio of the number of incident cases per year to the population of each year, multiplied by 100,000. Additionally, we calculated the mean dengue incidence rate for the period (2008–2018) for each neighborhood through the ratio of the mean number of incident cases in the study period to the mean population over the same period multiplied by 100,000 inhabitants.

**Figure 3:**
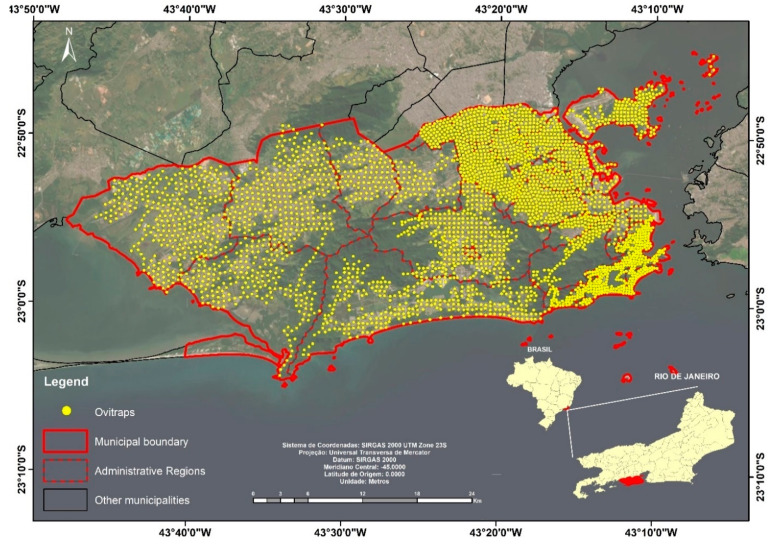
Map of the distribution of oviposition traps (ovitraps) in the city of Rio de Janeiro.

We calculated the *Aedes* EDI for each neighborhood from 2013 to 2014 through entomological monitoring by ovitraps. A total of 3400 ovitraps were distributed on a regular grid throughout the municipality with distances ranging from 300 to 600 m depending on the region of the city, covering all neighborhoods. Oviposition traps were installed monthly and collected after a period of 7 to 10 days (Figure 3).

### 2.4. Data Analysis 

We created dengue receptivity dimensions through principal component analysis (PCA) using the 17 indicators described in Table 1. PCA is a multivariate analysis technique that aims to transform the original, possibly correlated variables into components that are linear orthogonal combinations of these variables, to reduce the dimensions with the least possible loss of information [40]. The indicators were standardized, and we used the Kaiser criterion to identify the components to be selected, keeping only those with eigenvalues >1.0. The importance of each major component is assessed by the proportion of the total variance explained by the component. The load of each indicator was used to determine its importance in the component construction. In order to aid interpretation, components were named based on the indicators with the highest input.

We constructed the dengue receptivity index using multiple criteria analysis. This procedure involves map algebra, in which different information sheets are cross-referenced with their weights and scores, resulting in the synthesis map. Criteria must be standardized to carry out this integration, standardizing the units of all maps [41]. In this study, we used the components resulting from the principal component analysis as the information sheets to generate the dengue receptivity map. This map was generated by a multiple criteria analysis using a weighted linear combination method, in which each component was normalized and weighted according to its correlation index with the mean rate for the entire study period (2008 to 2018). Subsequently, the receptivity index was divided into quintiles, and the resulting map was constructed considering five classes (very high, high, medium, low, and very low). 

The dengue incidence rate and *Aedes* egg density values were interpolated by the weighted inverse distance weighting (IDW) method to generate a smooth and continuous layer from which isolines of equal value were extracted. The vector-format *Aedes* interpolated (smoothed) incidence rate and egg density layers were superimposed onto the principal component maps and the receptivity map for the visual examination of disease incidence and vector density associations against socio-environmental and territorial factors related to receptivity.

We used the Spearman correlation coefficient [42] to assess the relationship between dengue incidence, densities of the eggs and the components, and the dengue receptivity index.

All data construction and analysis procedures were performed using the R statistical software (R Foundation for Statistical Computing, Vienna, Austria) [43], and the spatial analysis and mapping procedures in the ArcGis 10.2 software (ESRI, Redlands, United States of America) [44].

## 3. Data Sources

### 3.1. Epidemiological Data

Data on notification of cases of dengue were obtained from the Notifiable Disease Information System (SINAN) of the Ministry of Health, from 2008 to 2018, aggregated at the neighborhood scale. We included in the analysis probable cases, confirmed by laboratory or clinical-epidemiological criteria, except those discarded by negative laboratory diagnosis.

### 3.2. Entomological Data

Data on *Aedes* eggs regarding mosquito infestation were obtained based on monitoring through oviposition traps (ovitraps) provided by the Municipal Health Secretariat of Rio de Janeiro from 2013 to 2014. These data show the number of *Aedes* eggs found at monitoring posts, which were aggregated by neighborhood, obtaining a mean of *Aedes* eggs by neighborhood.

### 3.3. Socioeconomic Data

The socioeconomic data were obtained from the 2010 Demographic Census of the Brazilian Institute of Geography and Statistics [24]. These data are made available according census tracts that were later aggregated by neighborhood for the construction of indicators.

### 3.4. Spatial Data

The city’s spatial and altimetry data were produced through laser mapping (Lidar, Rio de Janeiro City Hall) and were obtained from the Pereira Passos Institute [28]. The satellite images (Landsat 8 and Modis) were obtained from the International Research Institute for Climate and Society [34], Columbia University Earth Institute.

## 4. Results

Table 1 shows the data sources, calculation method, mean, and range of the indicators. Of the indicators we constructed, the following stand out: percentage of occupied area (Ind 01), ranging from 3.38% to 100%; net demographic density (Ind 03), ranging from 1372 inhabitants/km^2^ to 81,171 inhabitants/km^2^; percentage of subnormal agglomerates (Ind 04), with a mean of 11.18% and standard deviation of 14.7%; mean verticalization of buildings (Ind 06), with a mean of 6.23 m and standard deviation of 4.77 m; number of bordering neighborhoods (Ind 08), with a mean of 4.3 and standard deviation of 1.9; nighttime surface temperature (Ind 11), with a mean of 21.2 °C and standard deviation of 0.73 °C; and the percentage of streets not lined with trees (Ind 14), ranging from 0% to 77.28%. Indicators Ind 01, 03, 04, 06, and 14 showed high amplitude and dispersion. Ind 08 and 11 have a smaller amplitude and lower dispersion of data around the mean (Table 1). 

The PCA based on the 17 territorial indicators identified four components that explain 65.1% of the total data variance, divided as follows: component 1 (30.1%), component 2 (17.6%), component 3 (9.4%), and component 4 (8.0%) (Table 2).

**Figure 4:**
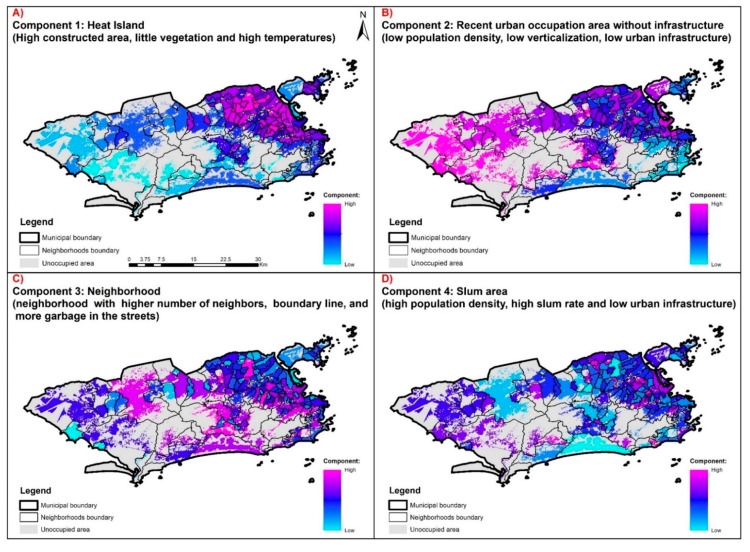
Spatialization of components from principal component analysis in Rio de Janeiro city: (**A**) component 1, (**B**) component 2, (**C**) component 3, and (**D**) component 4.

The indicators that most contributed to component 1 were Ind 01, Ind 02, Ind 10, and Ind 11 (direct relationship) and Ind 07 (inverse relationship) (Table 2). Component 1 represents areas of the territory with a high percentage of built-up area, low vegetation coverage, and high temperatures. As such, these are areas with a high density of residences and heat islands in the city. Their spatial distribution in the territory is uneven, with the highest values strongly concentrated in the north zone of the city and some neighborhoods in the west zone (Figure 4A).

Component 2 had the most substantial contribution from indicators Ind 14 and Ind 15 (direct relationship) and Ind 03 and Ind 06 (inverse relationship) (Table 2). This component represents areas of the territory with little urban infrastructure, low net population density, and low verticalization of buildings, that is, unconsolidated urban areas that were occupied more recently occupation and that lack infrastructure. Their spatial distribution is concentrated in the north and west zones of the city, with the former concentrating the largest number of neighborhoods with high values, while the latter includes presenting the largest areas with high values (Figure 4B).

The indicators that most contributed to component 3 were Ind 08, Ind 09, and Ind 16, all with a positive relationship (Table 2). Component 3 represents highly-connected neighborhoods within the city, with many neighbors and long border perimeters, and problems with garbage accumulation on the streets (Figure 4C). Neighborhoods with high component 3 values are concentrated around the city’s hills.

Component 4 comprised indicators Ind 03, Ind 04, Ind 16, and Ind 17, with a direct relationship with the component (Table 2). Component 4 describes the slum areas of the city, with a high residential area percentage, represented by subnormal agglomerates, with high net population density and a significant shortage of sanitation infrastructure. Their spatial distribution is highly heterogeneous, although the north zone has more neighborhoods with these characteristics (Figure 4D).

**Figure 5:**
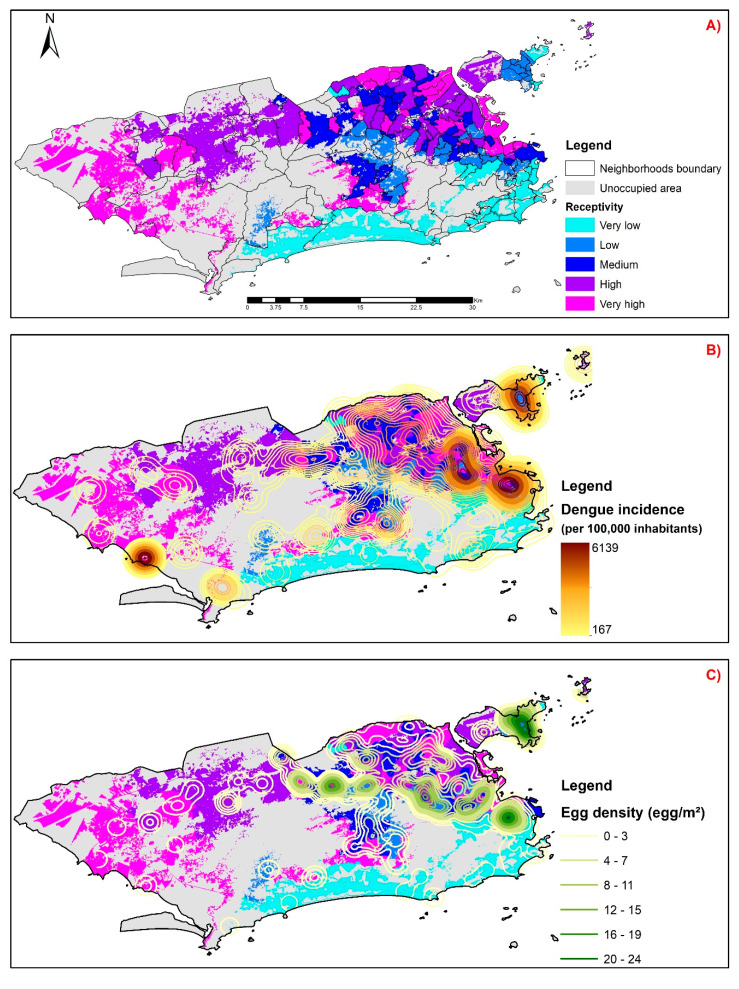
Map of dengue receptivity of Rio de Janeiro versus mean dengue incidence rate (2008 to 2018) and *Aedes* egg density index (2013–2014); (**A**) Mapping the receptivity index by neighborhoods in the city of Rio de Janeiro; (**B**) Mapping the density of the average incidence of dengue overlapping the receptivity index by neighborhoods in the city of Rio de Janeiro; (**C**) Mapping the density of *Aedes* eggs overlaid the receptivity index by neighborhoods in the city of Rio de Janeiro.

Figure 5A shows the dengue receptivity map resulting from cross-referencing the information sheets represented by the four components from the principal component analysis. We observed that the neighborhoods with the highest receptivity values are concentrated in the north and west zones of the city. The main areas with the least sensitivity were the south zone, center, and the west zone coastline (Figure 5A). 

The isolines representing the mean dengue incidence rate from 2008 to 2018 were overlaid onto the receptivity map, and we observed a high similarity in the spatial distribution of both. Dengue receptivity and mean incidence are spatially concentrated in the north and west zones, especially in the border region between them (Figure 5B). By analyzing the overlapping of isolines referring to the mean *Aedes* egg density for the 2013–2014 period, we also observed a similar spatial distribution to that of dengue-receptive areas. However, unlike what was observed for incidence, the areas with high of *Aedes* egg densities are more restricted to the north zone and the west–north boundary portion (Figure 5C).

The results of the Spearman correlation between the mean dengue incidence for the 2008–2018 period, and for each year, and the PCA components and the dengue receptivity index are shown in Table 3. The analysis of the mean dengue incidence throughout the study period revealed a 0.35 coefficient of correlation with receptivity. The highest correlation coefficient values were found in 2008, 2012, and 2016: 0.28, 0.44, and 0.47, respectively, which correspond to the epidemic years. Lower correlation values between receptivity index and dengue incidence rates were observed in non-epidemic years (2009, 2010, 2011, 2013, 2014, 2017, and 2018), especially when compared to the previous epidemic year with the same predominant serotype (Table 3). In the epidemic year 2008, there was a predominant circulation of serotype DENV-2; 2012 had the first epidemic with a predominance of DENV-4; 2016 had a predominant circulation of serotype DENV-2. We found a positive Spearman correlation of 0.21 between the receptivity index and the *Aedes* mean egg density for the overall study period.

In analyzing the correlation between components and outcomes, we observed that component 2 had the highest correlations with both dengue incidence and *Aedes* egg density (EDI), with even higher values than the receptivity index. The correlation coefficient between component 2 and the incidence rate for the 2008–2014 period was 0.37, while the correlation with the EDI for the 2013–2014 period was 0.28 (Table 3). For component 1, the year with the most significant correlation values was 2012, also an epidemic year.

## 5. Conclusions

The results of this study reveal four territorial dimensions: heat islands (component 1), unconsolidated urban sprawl area (component 2), urban connections (component 3), and slum area (component 4), which are urban processes that are related to dengue incidence and higher *Aedes* egg density. When analyzed in an integrated manner, these dimensions can stratify the territory of Rio de Janeiro into areas of greater receptivity to dengue.

Heat islands are urbanized areas, with low vegetation and high temperatures that are factors linked to the determination of dengue by providing good niches for the proliferation of *Aedes* vectors [36,45]. Unconsolidated urban sprawl areas are correlated with the dengue settlement process because they are spaces with a lack of urban infrastructure and services [37]. The neighborhoods with the greatest urban connections acting as nodes of the transport networks play an important role in the dissemination of epidemics, including dengue, due to the high circulation of people from different areas of the city [46]. As favelas in the city of Rio de Janeiro are areas with little in the way of urban infrastructure and services, low economic power, and high population density characteristics, these areas have a greater risk for dengue [37,38,47].

Unlike other cities, the area with the highest average temperatures in Rio de Janeiro, identified as a heat island, is not located in the center, but in the northern part of the city, isolated from the coast by two coastal massifs: Pedra Branca and Tijuca [48]. This region of the city has wide roads surrounded by obsolete or degraded urban areas, and little vegetation and high population density, summarized in component 1 [49].

The areas of unconsolidated urban growth, represented by component 2, are concentrated in the transition between the north and west zones of Rio de Janeiro. In this area, during the study period, we observed the highest incidence of dengue and most significant vector infestation obtained by larval index rapid Assay for *Aedes aegypti*—LIR*Aa* [50]. This component has the highest correlation with incidence and EDI, especially in the epidemic years. Thus, it is possible to associate dengue incidence and *Ae. aegypti* density with areas of unconsolidated urban sprawl lacking urban infrastructure [39,51].

Population flow and intra-urban spatial mobility, summarized in this article by the proximity between neighborhoods expressed in component 3, show that isolated neighborhoods have a lower tendency to maintain dengue epidemics, while the more connected neighborhoods with sub-center function in the city are more receptive to the disease [46].

Component 4 represents the area of the city occupied by slums. A city’s process of land use and occupation is closely linked to the conformation of particular places and landscapes within the urban territory.

Rio de Janeiro has undergone a complex process of urban sprawl with two well-defined main vectors: one responding to the economic elite, and the other responding to the working population [52]. The sprawl vector moved from the central region to the southern zone, and later to the west coast of the city, responding to the demands of the economic elites. The vector directed from the central region to the northern and inner zones of the west zone of the city responded to the demand of the popular segments. This region was the industrial and working area of the city [53] before Rio deindustrialization process observed after the 1980s. At the end of this process, these areas underwent a significant period of economic stagnation and decadence that is expressed in the territory to this day through a large amount of “roughness” present in these areas [25,52].

The stratification of the territory into areas of receptivity to dengue proposed by this study showed a spatial heterogeneity in their distribution in the municipality. The areas with the highest receptivity were concentrated in neighborhoods that are part of the “proletarian suburb” of the city, located in the transition areas between the north and west zones formed by railway and, later, road vectors. The less receptive areas were concentrated in the neighborhoods with the highest purchasing power and the best public and private services in general, which are located in the south zone of the city, the west zone coastline and the *Grande Tijuca* region in the north zone, the “*elite suburb*” [25,52].

Dengue and other diseases that have their determination influenced by the territory affect low-income populations. These populations live in places with less urban infrastructure and services, and worse environmental quality—not by choice, since these areas are the only ones that are available for this population group. The government should provide satisfactory conditions to eliminate or mitigate some of the determinants of the dengue health and disease process, while emphasizing that individual solutions do not have the capacity to change the territorial reality and consequently the determinants of dengue in each area [37,54,55].

The receptivity index was positively correlated with the dengue incidence rate for the 2008 and 2012 epidemic years and negatively correlated for the other years. This finding shows that dengue cases are concentrated in areas of greater receptivity at times when new virus types are introduced, when a large portion of the susceptible population is reached. In other low-incidence years, this trend is reversed, as these neighborhoods have reached a condition of collective immunity [33]. 

Epidemic years tend better to represent the spatial patterns of dengue occurrence, reducing the confounding effect of immunity acquired during previous epidemics. In 2012, the first DENV-4 dengue epidemic occurred in the city. That was the year with the highest correlations between incidence and components 1, 2, and the receptivity index. These higher correlations may be linked to the fact that, in 2012, the spatial distribution of dengue incidence corresponded more directly to territorial determinants, since the immunity factor was not present [56,57].

The concept of receptivity of socioenvironmental systems has been proposed in order to broaden our interpretation of current epidemiological profiles, thereby enabling a greater capacity to respond to the challenges arising from the change processes that take place in social and environmental contexts. These changes are expressed unevenly in the territory, being translated into different levels of receptivity for each portion of the inhabited space to be analyzed and different levels of vulnerability of the populations [58]. Territorialization occurs in the flow of knowledge and practices that are continuously constructed and reconstructed based on social dynamics [59]. It is necessary to approach the realities experienced by the subjects and the characterization of their territories to understand what makes them less or more receptive to a risk. This approach may assist in the search for alternatives for intervention and control of a health problem [60].

The receptivity index and its components were coherent with the socio-environmental and biological factors identified in the literature as determinants of dengue transmission in urban areas and can be used in other cities. This index can be used to target vector control and health services, strengthening policies and practices. However, this method cannot be used without taking into consideration the particularities of each city, because it is crucial that researchers describe each specific territory and propose indicators that portray the dimensions indicated by the components identified in this study, which may vary from city to city.

The evident limitations of dengue control programs, in the three spheres of government, regarding reducing or controlling the progression of dengue epidemics in a sustained manner over time, justify the search for methodologies that rationalize spending and optimize results. In this sense, the delimitation of priority areas for intervention (greater receptivity), proposed in this study, meets objectives that reduce the transmission of dengue, thereby optimizing spending resources. Furthermore, the delimitation of areas of greatest risk and greatest receptivity, and the locations and definitions of spatial patterns of the occurrence of dengue cases, can be used in the planning and development by the government for preventive actions and effective mitigators to combat this serious public health problem that the municipality faces.

In fact, arboviruses are a severe public health problem in Brazilian cities, and significantly challenge local health surveillance with highly heterogeneous transmission over both space and time [38,55,57,61]. The conclusions of this study reinforce the need for approaches that integrate the different dimensions of the inhabited space expressed in the territory to define areas receptive to dengue with the aim of bubbling up surveillance and intervention actions in the territory. [38,57,61]. In this context, the areas identified in this study as having the highest receptivity were defined in regions of dense and disorderly urban sprawl, with concentrations of socio-environmental processes related to dengue incidence and *Aedes* density. The stratification of priority areas for intervention actions by local surveillance integrated with the different dimensions of the territory optimizes resources and increases the understanding of health-disease processes.

Although the study proposed a purely spatial analysis, the use of demographic census data that are stationary in time and updated every 10 years ended up limiting the study, since territory is constantly changing due to the forces and flows that shape space. As a future perspective we have the idea of building indicators that reflect the conjuncture of the territory in a temporal way and using these in Baysian space-time models aiming at the identification of areas and periods of greater risk.

## Figures and Tables

**Table 1:** Territorial indicators, and their descriptive statistics and data sources.

Code	Indicator	Construction	Unit	Min–Max	Mean (SD)	Period	Source
Ind 01	Percentage of occupied area	Occupied Area/Total Area	%	3.38–100	76.013 (24.524)	2014	Municipality
Ind 02	Percentage of residential area	Residential Area/Total Area	%	0.74–100	64.878 (24.117)	2014	Municipality
Ind 03	Net demographic density	Total population/Residential area	inhab/km^2^	1372.74–81,171.23	16,778.461 (10,518.778)	2014	Municipality
Ind 04	Percentage of area of substandard clusters	Substandard Cluster Area/Residential Area	%	0–98.38	11.187 (14.7)	2014	Municipality
Ind 05	Percentage of are with strategic points	Strategic Points Area/Occupied Area	%	0–14.81	1.154 (2.547)	2014	Municipality
Ind 06	Mean verticalization	Total building heights/Total buildings	m	2.45–36.69	6.37 (4.778)	2014	Municipality
Ind 07	Percentage of vegetation	Vegetation Area/Total Area	%	0–94.34	25.108 (24.029)	2014	Municipality
Ind 08	Number of neighboring districts	Number of neighbors through neighborhood matrix	districts	1–13	4.375 (1.955)	2014	Own
Ind 09	Border perimeter with neighboring districts	Total length of boundary line	m	12.33–32048.3	7095.125 (4658.223)	2014	Own
Ind 10	Mean daytime surface temperature	Mean daytime temperature in the occupied area	°C	23.69–33.48	30.22 (2.623)	2008–2014	MODIS/IRI
Ind 11	Mean nighttime surface temperature	Mean nighttime temperature in the occupied area	°C	19.24–22.13	21.244 (0.733)	2008–2014	MODIS/IRI
Ind 12	Monthly cumulative rainfall	Mean cumulative rainfall in occupied area	mm^3^	85.13–153.31	100.058 (10.844)	2008–2014	Municipality
Ind 13	Percentage of households with unpaved streets	(v015 + v017 + v019/v001)—Spreadsheet surrounding01	%	0–63.63	4.831 (10.452)	2010	2010 Census
Ind 14	Percentage of households with no tree-lined streets	(v045 + v047 + v049/v001)—Spreadsheet surrounding01	%	0–77.28	19.869 (17.862)	2010	2010 Census
Ind 15	Percentage of households with streets without manholes	(v033 + v035 + v037/v001)—Spreadsheet surrounding01	%	0–91.96	12.342 (15.126)	2010	2010 Census
Ind 16	Percentage of households with streets with exposed trash	(v056 + v058 + v060/v001)—Spreadsheet surrounding01	%	0–40.34	4.038 (5.584)	2010	2010 Census
Ind 17	Percentage of households with streets with open sewage	(v050 + v052 + v054/v001)—Spreadsheet surrounding01	%	0–31.44	4.123 (5.534)	2010	2010 Census 2010

Ind: Indicator; Inhab/km^2^: Inhabitant per square kilometer.

**Table 2:** Factor loadings, eigenvalue, and explained variance of principal component analysis.

Indicators	Factor Loadings			
	Comp 1	Comp 2	Comp 3	Comp 4
Ind 01	0.91 *	0.03	−0.25	0.05
Ind 02	0.86 *	0.05	−0.18	0.06
Ind 03	0.26	−0.67 *	−0.03	0.52 *
Ind 04	0.05	-0.28	0.03	0.78 *
Ind 05	0.14	0.1	0.35	0.02
Ind 06	0	−0.67 *	0	−0.01
Ind 07	−0.88 *	0.02	0.25	−0.03
Ind 08	0.45	0.14	0.64 *	−0.16
Ind 09	0.28	0.41	0.50 *	−0.14
Ind 10	0.78 *	0.44	0.06	0.08
Ind 11	0.87 *	0.19	−0.03	0.05
Ind 12	−0.52	−0.52	0.31	0.03
Ind 13	−0.58	0.48	−0.34	0.09
Ind 14	−0.08	0.59 *	−0.22	0.25
Ind 15	−0.57	0.58 *	−0.33	0.1
Ind 16	−0.09	0.36	0.44 *	0.43 *
Ind 17	−0.29	0.52	0.28	0.39 *
Eigenvalue	5.11	2.99	1.6	1.36
% of variance	30.06	17.6	9.44	7.99

Ind: Indicator; Comp: Components; * Indicators with the greatest contribution in each component.

**Table 3:** Spearman correlation coefficient between receptivity index, dengue incidence rate, and *Aedes* egg density index (EDI) in the neighborhoods of Rio de Janeiro City.

	Incidence												EDI
Period	2008	2009	2010	2011	2012	2013	2014	2015	2016	2017	2018	2008–2018	2013–2014
Serotype	Denv 2	Denv 2	Denv 2	Denv 1	Denv 4	Denv 4	Denv 4	Denv 1	Denv 1	Denv 2	Denv 2		
Component 1	0.11	0.02	−0.15 *	−0.19 *	0.31 *	−0.13	0.03	0.27 *	0.20 *	0.12	0.12	0.12	0.07
Component 2	0.29 *	0.08	−0.21 *	0.24 *	0.41 *	−0.16 *	0.08	0.17 *	0.46 *	0.22 *	0.36 *	0.37 *	0.28 *
Component 3	0.04	−0.01	−0.01	−0.02	−0.02	−0.04	0.02	0.03	0.01	−0.16 *	−0.01	−0.05	−0.05
Component 4	0.14 *	0.02	−0.09	0.00	0.01	−0.12	−0.05	0.06	0.19 *	0.17 *	0.19 *	0.09	−0.06
**Receptivity**	0.28 *	0.08	−0.24 *	0.14	0.44 *	−0.22 *	0.05	0.23 *	0.47 *	0.30 *	0.39 *	0.35	0.21 *

* Significant with a significance level of 5%.
